# MAML1: a coregulator that alters endometrial epithelial cell adhesive capacity

**DOI:** 10.1186/s40738-021-00100-y

**Published:** 2021-03-27

**Authors:** Sadaf Zafir, Wei Zhou, Ellen Menkhorst, Leilani Santos, Evdokia Dimitriadis

**Affiliations:** 1 Department of Obstetrics and Gynaecology, University of Melbourne, Parkville, Victoria 3010 Australia; 2 Gynaecology Research Centre, Royal Women’s Hospital, Level 7, The Royal Women’s Hospital, 20 Flemington Road, Parkville, Victoria 3052 Australia

**Keywords:** Endometrial adhesion, Embryo implantation, Notch pathway, MAML1, Endometrial epithelial cell, Trophoblast cell, Hippo pathway

## Abstract

**Background:**

Abnormalities in endometrial receptivity has been identified as a major barrier to successful embryo implantation. Endometrial receptivity refers to the conformational and biochemical changes occurring in the endometrial epithelial layer which make it adhesive and receptive to blastocyst attachment. This takes place during the mid-secretory phase of woman’s menstrual cycle and is a result of a delicate interplay between numerous hormones, cytokines and other factors. Outside of this window, the endometrium is refractory to an implanting blastocyst. It has been shown that Notch ligands and receptors are dysregulated in the endometrium of infertile women. Mastermind Like Transcriptional Coactivator 1 (MAML1) is a known coactivator of the Notch signaling pathway. This study aimed to determine the role of MAML1 in regulating endometrial receptivity.

**Methods:**

The expression and localization of MAML1 in the fertile human endometrium (non-receptive proliferative phase versus receptive mid-secretory phase) were determined by immunohistochemistry. Ishikawa cells were used as an endometrial epithelial model to investigate the functional consequences of *MAML1* knockdown on endometrial adhesive capacity to HTR8/SVneo (trophoblast cell line) spheroids. After *MAML1* knockdown in Ishikawa cells, the expression of endometrial receptivity markers and Notch dependent and independent pathway members were assessed by qPCR. Two-tailed unpaired or paired student’s t-test were used for statistical analysis with a significance threshold of *P* < 0.05.

**Results:**

MAML1 was localized in the luminal epithelium, glandular epithelium and stroma of human endometrium and the increased expression identified in the mid-secretory phase was restricted only to the luminal epithelium (*P* < 0.05). Functional analysis using Ishikawa cells demonstrated that knockdown of *MAML1* significantly reduced epithelial adhesive capacity (*P* < 0.01) to HTR8/SVneo (trophoblast cell line) spheroids compared to control. *MAML1* knockdown significantly affected the expression of classical receptivity markers (*SPP1*, *DPP4*) and this response was not directly via hormone receptors. The expression level of Hippo pathway target Ankyrin repeat domain-containing protein 1 (*ANKRD1)* was also affected after *MAML1* knockdown in Ishikawa cells.

**Conclusion:**

Our data strongly suggest that MAML1 is involved in regulating the endometrial adhesive capacity and may facilitate embryo attachment, either directly or indirectly through the Notch signaling pathway.

**Supplementary Information:**

The online version contains supplementary material available at 10.1186/s40738-021-00100-y.

## Background

Implantation failure is a major contributor to infertility, contributing to more than 50% of the lost pregnancies [1, 2]. In order for successful implantation to occur, the endometrium must be receptive to a competent blastocyst [3]. Endometrial receptivity refers to the conformational and biochemical changes that take place in the functional layer of the endometrium to allow blastocyst attachment and invasion [4, 5]. It is known that endometrial tissue is only receptive to a competent blastocyst during the window of implantation which occurs during the mid-secretory phase (days 19–23 of a 28-day cycle) of the menstrual cycle and is otherwise refractory to blastocyst adhesion [4, 6]. Such cycle-dependent functional changes require delicate interplay between numerous hormones, cytokines and signaling pathways and are essential to secure a successful embryo attachment and implantation.

The Notch signaling pathway is one of the most highly conserved receptor-ligand signaling cascade families in multicellular organisms. It is involved in the regulation of cell proliferation, differentiation, invasion, adhesion and apoptosis via cell-cell interactions, thus directly impacting the fate of neighbouring cells [7, 8]. Notch receptors are plasma bound and have glycoprotein ligands. All four notch receptors and five ligands Jagged-1 and -2 and Delta-like (DLL)-1, − 3 and − 4 are known to be expressed by the endometrium [7, 9–12]. Notch pathway components are cyclically regulated in the endometrium [7] and Notch signaling plays an essential role in endometrial stromal cell decidualization and implantation [12–14]. Presence of Notch members in endometrial epithelium is of particular interest as it may suggest their role in facilitating the cell surface adhesive properties unique to a receptive endometrium and critical for successful implantation. In support of this hypothesis, conditional knockout of the Notch ligand JAG1 in mouse endothelial cells blocks endothelial-to-mesenchymal transition [15] which is a similar process compared to plasma membrane transformation that occurring in the endometrial luminal epithelium during receptive phase to facilitate embryo implantation [16]. Furthermore, it has been identified that Jagged1, Jagged2, DLL1 and Notch Receptor 1 (NOTCH1) expression are significantly lower at the mid-secretory phase in women with infertility, compared to fertile subjects [7, 17, 18]. However, the functional consequences of dysregulated Notch signaling on endometrial receptivity have not been clearly addressed.

Canonical Notch signaling is achieved when a Notch ligand on an adjacent cell membrane binds with a functional extracellular domain of the receptor on the target cell, resulting in release of the Notch intracellular domain (NICD). The NICD is then translocated to the nucleus where it can bind to a complex of proteins including recombining binding protein suppressor of hairless (RBPJ) and Mastermind Like Transcriptional Coactivator 1 (MAML1) to activate the transcription of Notch target genes [7, 8]. As one of the three co-regulators, MAML1 functions as a transcription coactivator for Notch pathway [19] thus directly regulating Notch signaling downstream activities. Studies have also shown that MAML1 expression levels in cells may act to alter Notch signaling, thus contributing to the diversity of functions resulting from Notch pathway [20, 21].

Apart from its well-known function in the Notch pathway, it has recently been identified that MAML1 also participates in the Hippo pathway. The Hippo signaling pathway is a highly conserved kinase cascade involved in the regulation of various biological processes including cell and tissue growth, cellular differentiation and regeneration [22]. Research has shown that members of the Hippo signaling pathway are expressed in the endometrium and are thought to play a role in decidualization and regulation of endometrial fibrosis [23, 24]. Activation of Hippo pathway phosphorylates either the mammalian transcriptional activator ‘Yes-associated protein’ (YAP), or its paralog with PDZ-binding motif (TAZ) which encourages cytoplasmic retention and prevents YAP/TAZ’s transport into the nucleus. When the hippo cascade is inactive, unphosphorylated YAP/TAZ can translocate into the nucleus to mediate gene transcription [22]. Most recently, a study has shown that MAML1/2 are involved in the retention of YAP/TAZ in the nucleus and promoting transcriptional activity [25]. This suggests that MAML1 may play an important role in the regulation of Hippo signaling and could be involved in the Hippo pathway in the endometrial epithelium and therefore involved in endometrial receptivity. MAML1 can regulate other signaling pathways including Sonic Hedgehog and NF-kB pathways [26]. It has been shown that NF-kB pathway can regulate Hippo signaling targets such as ANKRD1 in a Hippo signaling-independent manner [27], illustrating the complexity of downstream effects regulated by MAML1.

Loss of MAML1 in mice results in growth retardation, skeletal degeneration and postnatal lethality [28] so its endometrial function including uterine receptivity is unknown. To the best of our knowledge, there is presently no research exploring the function of MAML1 on endometrial receptivity. This study aimed to determine the role of MAML1 in regulating endometrial epithelial cell adhesive capacity and understand its mechanism of action through either Notch dependent or independent pathways.

## Methods

### Endometrial tissue collection

Following approval from the Human Research Ethics Committees at Monash Health (#03066B) and the Royal Women’s Hospital (SSA1813), functional layer endometrial tissue biopsies were collected from women (aged 26–42 years) who provided informed consent prior to undergoing surgical procedures for mirena insertion or benign ovarian cyst assessment. All women in this study were fertile with proven parity (≥1 parous pregnancy), regular menstrual cycles (28-32 days) and were not using intrauterine or hormonal contraceptives for at least 3 months before surgery. Tissue samples were collected by curettage and examined by gynecological pathologists at the Royal Women’s Hospital to confirm cycle stage. Proliferative phase (*n* = 4) and mid-secretory phase (*n* = 4) endometrium were used for this study.

### Antibodies and cell lines

Rabbit polyclonal antibody against MAML1 (PA5111083) was purchased from Thermo (Waltham, MA, USA). HRP-GAPDH antibody was from Cell Signaling Technology (Danvers, MA, USA, #3683). IgG isotype control (Rabbit IgG fraction, #X0903) was from DAKO (Glostrup, Municipality, Denmark). Ishikawa and HTR8/SVNeo cells were used to investigate the adhesive capacity of the endometrium and blastocyst-endometrium interactions. The Ishikawa cell line was provided by Dr. M. Nishida (Tsukuba University, Tochigi, Japan). The Ishikawa cell line is derived from endometrial adenocarcinoma cells and represents a similar phenotype to endometrial luminal and glandular epithelial cells, not only in the expression of estrogen and progesterone receptors (ESR and PGR) but also in its cell-cell adhesive capacity [29, 30]. The HTR8/SVneo trophoblast cell line (CRL-3271) was purchased from the ATCC and cultured as in the manufacturer’s instructions.

### *MAML1* siRNA transfection

Ishikawa cells at 70–80% confluency were transfected with Lipofectamine RNAiMAX and Opti-MEM medium (Thermo) containing 20 nM *MAML1* siRNA or scrambled control (Dharmacon, Lafayette, CO, USA) according to the manufacturer’s instructions. After 24 h the transfection medium was replaced with fresh culture medium and transfected Ishikawa cells were cultured for 48 h before being subjected to spheroid adhesion assay or other downstream analyses.

### Immunohistochemistry

Human endometrial tissues were fixed in 10% formalin, embedded in paraffin and sectioned at 4 μm thickness. Slides were dewaxed, rehydrated and subjected to heat induced antigen retrieval (microwaving in 10 mM sodium citrate pH 6 for 6 min). Endogenous peroxidase activity was then blocked using 3% hydrogen peroxide diluted in methanol and incubated for 15 min at room temperature. After Tris-buffered saline (TBS) wash (twice for 5 min each), slides were incubated in a non-immune blocking buffer (10% goat serum and 2% human serum in TBS) for 30 min at room temperature. Immediately following this MAML1 antibody (1 μg/mL) and a normal rabbit immunoglobulin fraction (1 μg/mL) were added and incubated overnight at 4 °C. These slides were then washed with TBS-Tween 0.6% (v/v) and positive signaling was revealed via the avidin-biotin- diaminobenzidine system. Sections were counterstained with hematoxylin to indicate cell nuclei (blue). Slides were then mounted with DPX and imaged using an Olympus light microscope. Staining intensity scores were determined by two individual scorers blinded to the patient characteristic and averaged for statistical analysis, as previously described [31]. Briefly, a score of 0 denoted no positive MAML1 staining and 3 was intense staining. Each score was based on overall staining intensity of the whole endometrial section.

### Spheroid adhesion assay

Passaged HTR8/SVneo cells were counted using Countess and 2000 cells/well were plated into wells of a U-shaped, ultra-low attachment 96 well plate (Sigma, CatCLS7007, St. Louis, MO, USA). Cells were then cultured for 48 h to form spheroids and 20 spheroids were collected and transferred to transfected Ishikawa cell monolayer (20 spheroids/well of 96 well plate) to initiate spheroid adhesion assay. The initial spheroid numbers per well were recorded using a light microscope before being allowed to co-incubate for 4 h at 37 °C. Afterward, all culture medium was removed and each well was gently washed once by adding 150 μL PBS to remove non-adherent spheroids. The remaining spheroids were then counted, and the percentage attachment was expressed as a percentage of the original spheroid number as previously reported [32, 33].

### RNA isolation and RT-qPCR

RNA was retrieved from transfected Ishikawa cells. Cells were lysed with TRI Reagent (Sigma). RNA was isolated according to the manufacturer’s protocol and treated with RNase-free DNase set (Qiagen) to remove genomic DNA contamination. RNA concentration was determined by Nanodrop spectrophotometers (Thermo). After isolation, 300 ng total RNA was converted to cDNA using SuperScript™ III First-Strand Synthesis System (18080–051, Thermo). qPCR was performed on the Applied Biosystems ViiA7 system using SYBR Green Master Mix (4,367,659, Thermo) as previously described [34]. Primers used are summarized in Additional file 1. Gene expression was normalized to *18S*. Relative expression levels were calculated using the comparative cycle threshold method (ΔΔCt).

### Immunoblotting

Protein was extracted from the organic phase of TRI Reagent based RNA isolation as previously described [35]. Proteins were resolved by SDS-PAGE (150 V, 1 h) and transferred to polyvinylidene difluoride membranes (pre-soaked with methanol). Membranes were blocked with 5% skim milk in TBS for 1 h and incubated with MAML1 antibody (1:1000) prepared in 5% skim milk. Membranes were washed three times with TBS-Tween 0.1% (v/v) and incubated with horse radish peroxidase (HRP)-conjugated secondary antibody against rabbit and HRP-GAPDH (13,000, as a loading control). Labeled proteins were detected by chemiluminescence. For quantification, appropriate bands were assessed by densitometry, normalized against a loading control GAPDH, as previously described [36].

### Statistics

Statistical analysis was performed using PRISM 8.0 and two-tailed paired or unpaired student’s t-test were used for statistical analysis with a significance threshold of *P* < 0.05. Data were presented as the mean ± SEM.

## Results

### MAML1 expression significantly increases in the luminal epithelium during the receptive mid-secretory phase compared to the non-receptive proliferative phase in fertile women

**Fig. 1:**
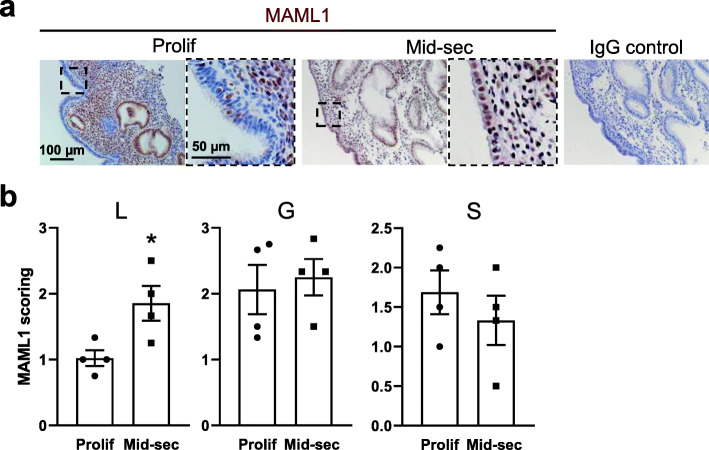
Comparison of MAML1 expression in fertile proliferative phase and mid-secretory phase endometrium. **a** Immunohistochemistry detection of MAML1 in luminal epithelium (L) glandular epithelium (G) and stroma (S) of human endometrium. A nuclear labeling was recorded in all cell types. The specificity of MAML1 labeling was confirmed through the inclusion of an isotype control in which the non-immune antibody of the same isotype was substituted for the MAML1 antibody at the same concertation. Sections were counterstained with hemotoxylin to highlight the cell nuclei (blue). **b** Staining intensity of MAML1 was semi-quantitated by scoring staining in tissues blinded to cycle stage. Data were presented as mean ± SEM. (*n* = 4). *P < 0.05

We first sought to determine the relevance of MAML1 on receptivity by assessing its expression and immunolocalization on proliferative phase and mid-secretory phase endometrium, which respectively represent non-receptive and receptive status of the endometrium for an implanting blastocyst. Immunohistochemistry staining illustrated a nuclear localization of MAML1 protein in the luminal epithelium, glandular epithelium and stroma of the endometrium of fertile women during the proliferative phase and mid-secretory phase (Fig. 1a). The intensity and frequency of MAML1 was scored by two blinded scorers independently on a scale of 0–3. Only luminal epithelium showed a significant increase (*P* < 0.05) of MAML1 staining in the mid-secretory phase when compared to the proliferative phase (Fig. 1b). No significant differences were recorded in either glandular epithelium or stroma between two phases (Fig. 1b). The specificity of MAML1 antibody labeling was confirmed through the inclusion of an IgG control in which MAML1 antibody was replaced with a normal rabbit immunoglobulin fraction (Fig. 1a).

### siRNA knockdown of *MAML1* in Ishikawa cells impaired HTR8/SVneo spheroid adhesion

**Fig. 2:**
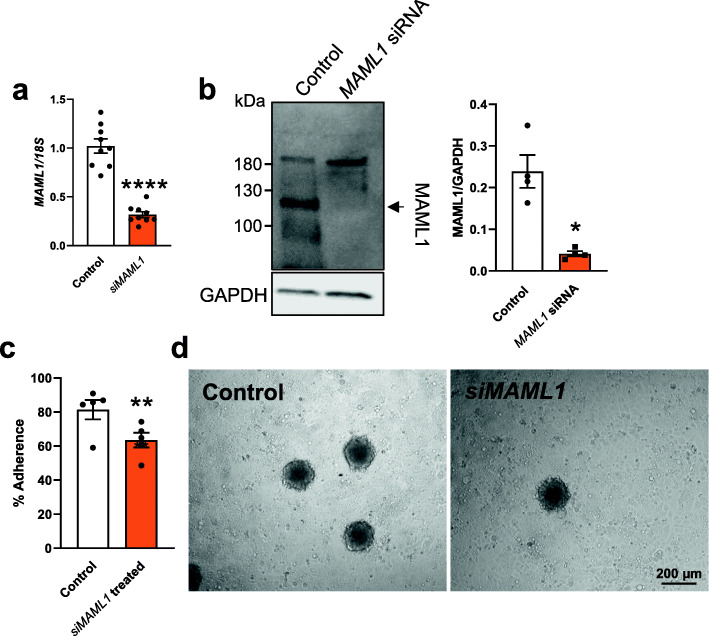
Examination of the effect of *MAML1* knockdown on Ishikawa cell adhesive capacity. Ishikawa cells were transfected with either *MAML1* siRNA (20 nM) or scrambled control (20 nM) before HTR8/SVneo spheroid adhesion assay or other analysis. **a** *MAML1* knockdown was confirmed by qPCR. Expression levels were normalized to *18S* (*n* = 9). **b** MAML1 knockdown was confirmed by immunoblotting and densitometry, normalized against a loading control GAPDH (*n* = 4). **c** *MAML1* knockdown significantly compromised the spheroid adhesion compared to scrambled control (*n* = 5). **d** Representative images are presented to show attached spheroids on the Ishikawa cell monolayer after adhesion assay. Data were presented as mean ± SEM. *P < 0.05, **P < 0.01, ***P < 0.001

Ishikawa cells (endometrial adenocarcinoma cells) were used as a model to assess the functional consequences of *MAML1* knockdown on epithelial cell adhesion. Ishikawa cells were transfected with 20 nM of scrambled control or *MAML1* siRNA and subjected to spheroid adhesion assay to assess changes of adhesive capacities. qPCR analysis confirmed a successful knockdown of *MAML1* in *MAML1* siRNA treated group, compared to scrambled control (*P* < 0.0001, Fig. 2a). Such knockdown was further validated at the protein level by immunoblotting and densitometry analysis (*P* < 0.05, Fig. 2b). HTR8/SVneo spheroids added to cultured Ishikawa cell confluent monolayers in a spheroid adhesion assay showed the functional consequences of *MAML1* knockdown on the adhesion of spheroids to the Ishikawa cells. Figure 2c and d demonstrated that HTR8/SVneo spheroid adhesion was significantly lower in siRNA *MAML1* transfected Ishikawa cells compared to the scrambled control transfected Ishikawa cells (*P* < 0.01).

### The effect of *MAML1* knockdown on notch dependent and independent pathway members

**Fig. 3:**
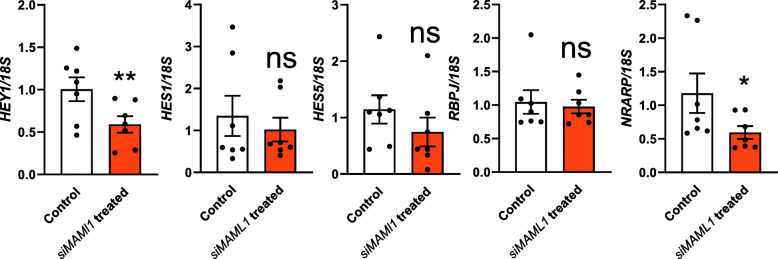
Qualitative qPCR examination of the effect of *MAML1* knockdown on common Notch pathway members. *HEY1* and *NRARP* expression were significantly decreased after *MAML1* knockdown in Ishikawa cells compared to scrambled control. Expression levels were normalized to *18S* (*n* = 7). Data were presented as mean ± SEM. *P < 0.05, **P < 0.01, ns: no significant difference

We next determined if knockdown of *MAML1* in Ishikawa cells affected Notch pathway dependent activities. Hairy and enhancer of split (HES) 1, HES5 and Hairy/enhancer-of-split related with YRPW motif protein 1 (HEY1) are most commonly used to investigate Notch pathway transcriptional activity [7, 37] and were therefore chosen for qPCR analysis. Expression levels of *RBPJ*, the MAML1 coactivator and Notch Regulated Ankyrin Repeat Protein (*NRARP*), downstream effector of Notch pathway were also examined. As shown in Fig. 3, when comparing *MAML1* siRNA treated to scrambled control treated Ishikawa cells, *HEY1* and *NRARP* expression were significantly decreased following *MAML1* knockdown. The difference in expression levels of *HES1* and *HES5* in *MAML1* siRNA treated cells compared to scrambled control treated cells, were not statistically significant. Similarly, *RBPJ*, a coactivator of MAML1 showed no significant difference in expression after *MAML1* knockdown (Fig. 3).

**Fig. 4:**
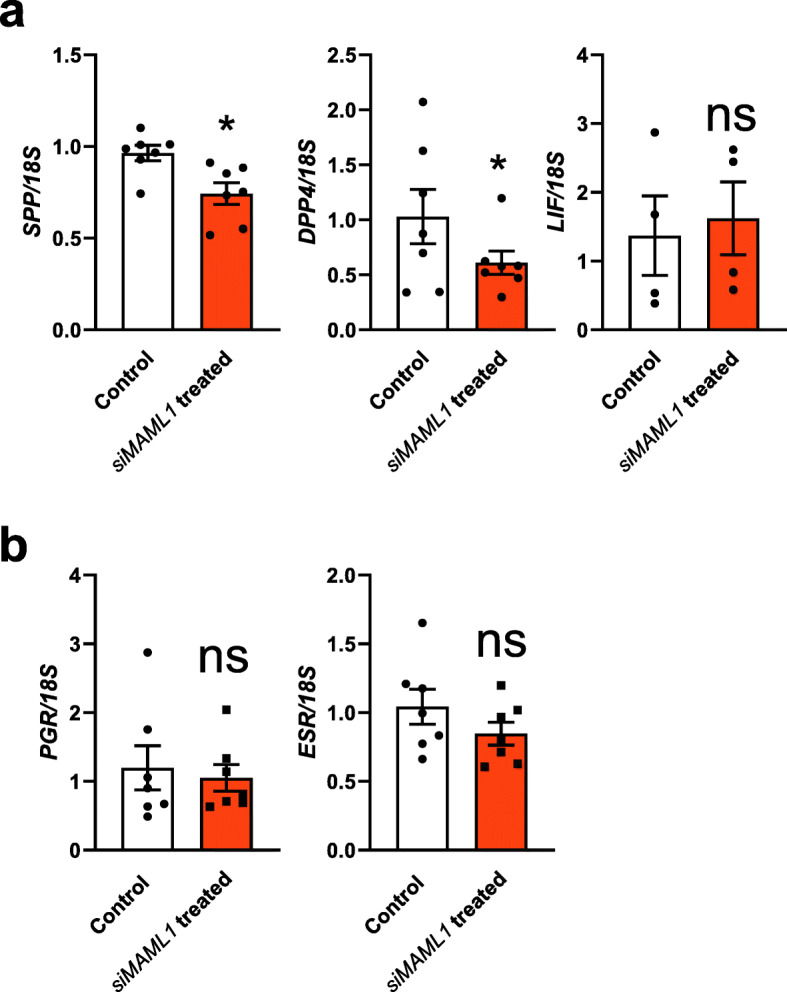
Effect of *MAML1* knockdown on the expression of endometrial receptivity markers and hormone receptors. Expression levels were normalized to *18S* (*n* = 7). Data were presented as mean ± SEM. *P < 0.05, ns: no significant difference

As *MAML1* siRNA treatment compromised Ishikawa adhesive capacity, we next selected three endometrial receptivity markers namely SPP1, Dipeptidyl peptidase 4 (DPP4) and LIF [38] and examined their mRNA expressional changes after *MAML1* knockdown. qPCR examination showed that the expression of *SPP1* and *DPP4* were significantly decreased (*P* < 0.05) while for *LIF* no significant difference was observed in MAML1 siRNA treated Ishikawa cells compared to scrambled control treated cells (Fig. 4a). We further confirmed that such changes were not mediated through the *ESR* and *PGR* as no significant changes were recorded of these two hormone receptors between control and MAML1 siRNA treated groups (Fig. 4b).

**Fig. 5:**
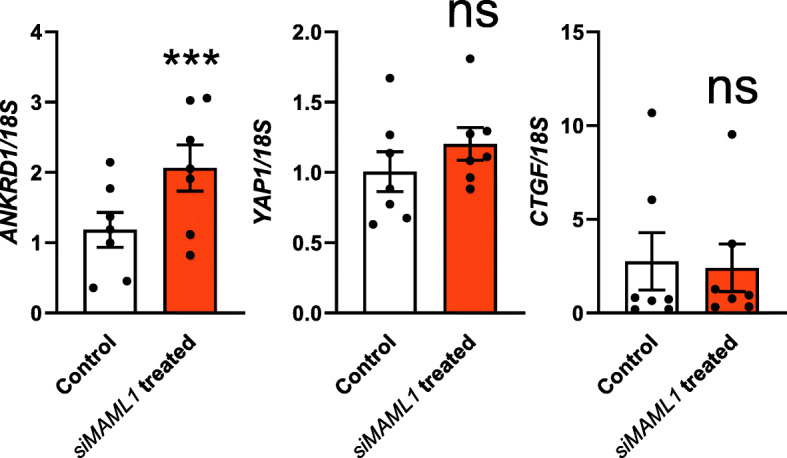
Examination of the effect of *MAML1* knockdown on the expression of Hippo pathway downstream effectors. Expression levels were normalized to *18S* (*n* = 7). Data were presented as mean ± SEM. ***P < 0.001, ns: no significant difference

The expression levels of Hippo transcriptional targets, *ANKRD1* and Connective tissue growth factor (*CTGF*), and a downstream regulator, *YAP1* were also examined after *MAML1* siRNA treatment in Ishikawa cells. Most notably, *ANKRD1* expression was significantly higher (*P* < 0.001) in *MAML1* siRNA treated Ishikawa cells compared to control (Fig. 5). *YAP1* expression increased as well, though not significant (*P* > 0.05), while *CTGF* expression remained largely unchanged between groups (Fig. 5).

## Discussion

This study identifies a novel role for MAML1 in endometrial receptivity via its regulation of endometrial epithelial cell adhesive capacity. We demonstrated that MAML1 was upregulated in the endometrial luminal epithelium during the mid-secretory (receptive) phase of the menstrual cycle and that loss of MAML1 impaired trophoblast (HTR8/SVneo) spheroid attachment to an endometrial epithelial cell monolayer in vitro. Our data suggests MAML1 signaling may be required for endometrial epithelial expression of key regulators of endometrial receptivity including SPP1 and DPP4.

Overall, this study strongly supports a role for MAML1 in regulating endometrial receptivity. In fertile endometrium we found MAML1 production was significantly increased in the luminal epithelium during the receptive phase and using an in vitro model of blastocyst implantation [34] we demonstrated that endometrial epithelial MAML1 production was required for trophoblast spheroid adhesion. While this study only included a few clinical samples from the proliferative and mid-secretory phases to represent non-receptive and receptive status of the endometrium, it nevertheless supports the importance of MAML1 in regulating human endometrial receptivity. Further studies using larger samples sizes and in tissues from women with fertility and infertility/implantation failure are required to confirm our observations. Using qPCR we identified that MAML1 positively regulates Ishikawa cell production of known regulators of endometrial epithelial receptivity, DPP4 and SPP1 [39–43]. By meta-analyses, both SPP1 and DPP4 are elevated in the endometrium during the receptive phase [40, 44]. SPP1 and DPP4 are both membrane-bound extracellular glycoproteins which have established roles in cell adhesion. SPP1 is maximally produced by endometrial glands and found in uterine lavage during the mid-secretory phase [45, 46]. SPP1 is the only gene consistently up-regulated during the receptive phase in analyses of five studies [47], highlighting its likely importance in regulating receptivity. The role of SPP1 in regulating embryo adhesion has been well characterized in pigs, where SPP1 is secreted by the uterus just prior to embryo implantation and promotes embryo adhesion by interaction with integrins on the conceptus-maternal interface [48]. Like SPP1, DPP4 shows up-regulation during the receptive phase in both mRNA and immunohistochemical studies [47, 49, 50]. DPP4 is predominantly expressed by the uterine luminal and glandular epithelium [50] and is down-regulated in women with repeated implantation failure [49]. There has been no previous investigation of direct MAML1 regulation of either SPP1 or DPP4, however MAML1 is predicted to directly regulate transcription of both SPP1 and DPP4 by the presence of RBPJ binding sites in the promoter region of each gene and SPP1 is a direct target of Notch signaling [51, 52].

MAML1 has largely been described as a co-activator of the Notch pathway [53]. Here we found that MAML1 activity in Ishikawa cells was required for transcription of multiple Notch pathway factors including *HEY1*, *NRARP* and *SPP1*. *HEY1* is regulated by MAML1 in melanoma [54] and human umbilical venous endothelial cells [55]. NRARP is a negative regulator of Notch signaling [56] and in Xenopus, the NRARP complex can include Mastermind [57]. *SPP1* is a direct target of the Notch pathway in osteoblasts [51] and human hepatocellular carcinoma [52]. Interestingly, MAML1 loss had no effect on *HES1 or HES5* expression in Ishikawa cells. It is possible that MAML3 is acting to compensate for loss of MAML1 in this study given their structural and functional similarity to MAML1: studies in mice suggest that Maml1/3 may perform redundant roles during embryogenesis with a duel knockout required to replicate pan-notch defects [20]. In mouse embryos loss of both Maml1 and Maml3 is required to alter transcription of *Hey1* and *Hes5* [20]. All together, these data support a direct role for MAML1 in the regulation of Notch signaling in Ishikawa cells although the functional effect of MAML2/3 in compensating for the loss of MAML1 requires further investigation.

Despite the Notch family first being identified as cell adhesion molecules in Drosophila and evidence demonstrating this function is conserved in mammals [58], there is a paucity of research investigating the mechanism by which the Notch signaling pathway mediates cell adhesion [58]. Notch signaling promotes the transcription and activation of cell adhesion molecules including integrins [58]. We have previously shown that Notch 1, DLL1 and Jagged1 are all significantly decreased in the endometrial epithelium of women with unexplained primary infertility [7, 18]. Interestingly, loss of endometrial epithelial DLL1 in women with unexplained primary infertility may be due to excess cleavage of DLL1 by ADAM17, increasing soluble (s)DLL1 in the uterine lumen [59]. sDLL1 is increased in the uterine lavage of women with primary infertility and impairs blastocyst adhesion in our in vitro model of blastocyst implantation [59]. It would be interesting to determine whether Notch pathway components sDLL1 or Jagged1, which are downregulated in unexplained primary infertility, alter MAML1 activity in endometrial epithelial cells – certainly a mutation in the DLL1 gene which is predicted to impair DLL1 binding to Notch receptors down-regulates *MAML1* (and *HEY1)* in peripheral blood mononuclear cells [60].

MAML1 also has reported functions independent of the Notch pathway [61], including the Hippo [25], Sonic Hedgehog and NF-kB pathways [26]. We explored whether MAML1 regulated Hippo pathway components in Ishikawa cells. There is little information on the role of the Hippo pathway in the endometrium. The Hippo/YAP pathway is required for decidualization of endometrial stromal cells [23], likely associated with the Hippo pathway’s role in regulating cell proliferation to control organ size [25]. ANKRD1 and CTGF are commonly considered as Hippo pathway transcriptional gene targets. While CTGF expression remained unchanged, loss of MAML1 significantly increased ANKRD1 transcription here suggesting that MAML1 may act to turn the Hippo pathway ‘on’ in endometrial epithelial cells, which would inhibit transcription. However, this is inconsistent with previous research which demonstrated that MAML1 facilitates nuclear retention of YAP/TAZ (Hippo pathway ‘off’), and therefore, promotes transcription [25]. Moreover, DPP4 has been identified as a target of the Hippo/YAP pathway in cardiac fibroblasts [62] and was positively regulated by MAML1 in Ishikawa cells here. Possibly MAML1 is regulating ANKRD1 in a Hippo-independent manner – ANKRD1 transcription is repressed by sonic hedgehog treatment in mouse fibroblasts [63] and NF-kβ inhibition in human airway smooth muscle cells [27]. Altogether these studies suggest that the MAML1 may act independently of the Notch pathway in the endometrial epithelium, but further research is required to determine the precise role of MAML1 in endometrial receptivity.

## Conclusions

In conclusion, our study has provided the first evidence that MAML1 may be a critical component in the regulation of endometrial epithelial cell adhesive capacity during endometrial receptivity and blastocyst implantation. Our study suggests that MAML1 may be acting as a co-activator of the Notch signaling pathway to directly or indirectly alter expression and, perhaps, function of endometrial receptivity markers. We also show that MAML1 may have non-canonical actions in the endometrial epithelium, including acting as a co-activator of Hippo signaling. Understanding the mechanism of action of MAML1 in the endometrium may uncover novel targets for therapies to treat female infertility and key pathways indicating the optimal time for embryo transfers to improve IVF success.

## Supplementary Information

**Additional file 1.** Primers used throughout this study.

## Data Availability

All data generated through this study are included in this article.

## Abbreviations

MAML1: Mastermind Like Transcriptional Coactivator 1
ANKRD1: Ankyrin repeat domain-containing protein 1
DLL: Delta-like
NOTCH1: Notch Receptor 1
NICD: Notch intracellular domain
YAP: Yes-associated protein 1
RBPJ: Recombining binding protein suppressor of hairless
PGR: Progesterone receptor
ESR: Estrogen receptor
TBS: tris-buffered saline
PBS: phosphate-buffered saline
HEY1: Hairy/enhancer-of-split related with YRPW motif protein 1
HES1: Hairy and enhancer of split 1
HES5: Hairy and enhancer of split 5
NRARP: Notch Regulated Ankyrin Repeat Protein
SPP1: Secreted Phosphoprotein 1
DPP4: Dipeptidyl peptidase 4
LIF: Leukemia Inhibitory Factor
CTGF: Connective tissue growth factor
HRP: Horse radish peroxidase
