# Supplementary material for: MAML1: a coregulator that alters endometrial epithelial cell adhesive capacity
Source: Fertil Res Pract. 2021 Mar 27;7:8. doi: 10.1186/s40738-021-00100-y (PMC8004388; doi:10.1186/s40738-021-00100-y)
Supplement: Supplementary file 1 — Additional file 1. Primers used throughout this study. [file 40738_2021_100_MOESM1_ESM.docx]

| **Additional file 1. Primers used throughout this study.** | | |
| --- | --- | --- |
| **Gene** | **Forward (5'-3')** | **Reverse (5'-3')** |
| *MAML1* | CCCCAGTGAGTCATTTCCTCT | GAGGTTGCTTTGCGATATGGA |
| *HEY1* | GTTCGGCTCTAGGTTCCATGT | CGTCGGCGCTTCTCAATTATTC |
| *HES1* | TCAACACGACACCGGATAAAC | GCCGCGAGCTATCTTTCTTCA |
| *HES5* | AAGCACAGCAAAGCCTTCGT | CTGCAGGCACCACGAGTAG |
| *RBPJ* | CTGACTCAGACAAGCGAAAGC | AGGAACACACCAATGTCATCAC |
| *SPP1* | ACAGCCAGGACTCCATTGAC | ACACTATCACCTCGGCCATC |
| *DPP4* | TACAAAAGTGACATGCCTCAGTT | TGTGTAGAGTATAGAGGGGCAGA |
| *LIF* | TGAACCAGATCAGGAGCCAAC | CCACATAGCTTGTCCAGGTTG |
| *PGR* | ACCCGCCCTATCTCAACTACC | AGGACACCATAATGACAGCCT |
| *ESR* | GGGAAGTATGGCTATGGAATCTG | TGGCTGGACACATATAGTCGTT |
| *YAP1* | TAGCCCTGCGTAGCCAGTTA | TCATGCTTAGTCCACTGTCTGT |
| *CTGF* | CAGCATGGACGTTCGTCTG | AACCACGGTTTGGTCCTTGG |
| *ANKRD1* | CGTGGAGGAAACCTGGATGTT | GTGCTGAGCAACTTATCTCGG |
| *NRARP* | TCAACGTGAACTCGTTCGGG | ACTTCGCCTTGGTGATGAGAT |
| *18S* | GATCCATTGGAGGGCAAGTCT | CCAAGATCCACCTACGAGCTT |
